# Liquidity index of the Lublin loess as a function of cone resistance *q*_c_ from CPTU test

**DOI:** 10.1038/s41598-025-94629-3

**Published:** 2025-03-22

**Authors:** Krzysztof Nepelski, Agnieszka Lal, Monika Krzysiak, Izabela Skrzypczak, Piotr Ochab, Wojciech Gosk

**Affiliations:** 1 Faculty of Civil Engineering and Architecture, Lublin University of Technology, ul. Nadbystrzycka 40, Lublin, 20-618 Poland; 2 GeoNep Geotechnika, ul. Wigilijna 4, Lublin, 20-502 Poland; 3 Faculty of Civil and Environmental Engineering and Architecture, Rzeszow University of Technology, ul. Poznańska 2, Rzeszów, 35-084 Poland; 4 Faculty of Civil Engineering and Environmental Sciences, Bialystok University of Technology, ul. Wiejska 45E, Białystok, 15-351 Poland

**Keywords:** Loess, Liquidity index, Cone resistance, CPTU, Correlation, Civil engineering, Characterization and analytical techniques

## Abstract

The paper presents the results of the calibration of an interpretive formula for the determination of *I*_L_ based on cone resistance *q*_c_ for loess soils. The studies were carried out on characteristic soils that occur in the Lublin region. For this purpose, three CPTU static soundings were selected in which a representative soil profile with a total length of 21 m was extracted. Boreholes were drilled in close vicinity, at distances ranging within 1.0 m, and samples for laboratory testing were collected at intervals of 0.25 m. A total of 86 samples were tested and the liquidity index was determined. The *I*_L_ values specified in the laboratory were paired with the cone resistances *q*_c_ averaged over the depth range corresponding to the sampling. The *q*_c_/p_a_ – *I*_L_ distribution was then analysed and a formula correlating both parameters was derived. The cone resistance depends not only on water content but also on a number of other factors, therefore, the estimation of *I*_L_ from CPTU tests should be regarded as an approximation, while an accurate identification of the subsoil requires further investigations. Nevertheless, such information provides valuable knowledge in the initial stages of subsoil recognition, e.g. during design work.

## Introduction

In civil engineering, the subsoil is most often treated as an elasto-plastic medium with spatially variable physical and mechanical properties^1^. The geotechnical parameters that determine its bearing capacity and suitability for foundation setting depend on many factors and tend to vary over time. In the identification of soil parameters, apart from the selection of an appropriate testing methodology, it is also essential that the number of results is sufficient to allow statistical evaluation and thus minimize the impact of errors made during the testing process. Furthermore, it is important to obtain data using rapid methods, for which in situ tests provide an excellent tool. In addition, the combination of fast and data-rich in situ testing with GIS analysis enables the proper assessment of the suitability of a site for construction purposes already at the earliest stages of investment or land use planning^2^. This is particularly the case in areas of high urbanisation.

One of the most important aspects that significantly influences the bearing capacity of fine-grained soils is their water content, which decides, among other factors, their consistency. The initial step to determine soil consistency is macroscopic testing, which provides only an approximate result. For classification purposes, a numerical parameter in the form of the liquidity index (*I*_L_) is used. The liquidity index is the relative moisture content, determined on the basis of the natural water content and liquid and plastic limits from the relationship:

1 $$\:{I}_{L}=\frac{\text{W}-{P}_{\text{L}}}{{L}_{\text{L}}-{P}_{\text{L}}}$$

where: $\:\text{W}$ – natural water content [%],$\:\:{P}_{\text{L}}$– plastic limit [%], $\:{L}_{\text{L}}$ – liquid limit [%].

**Fig. 1:**
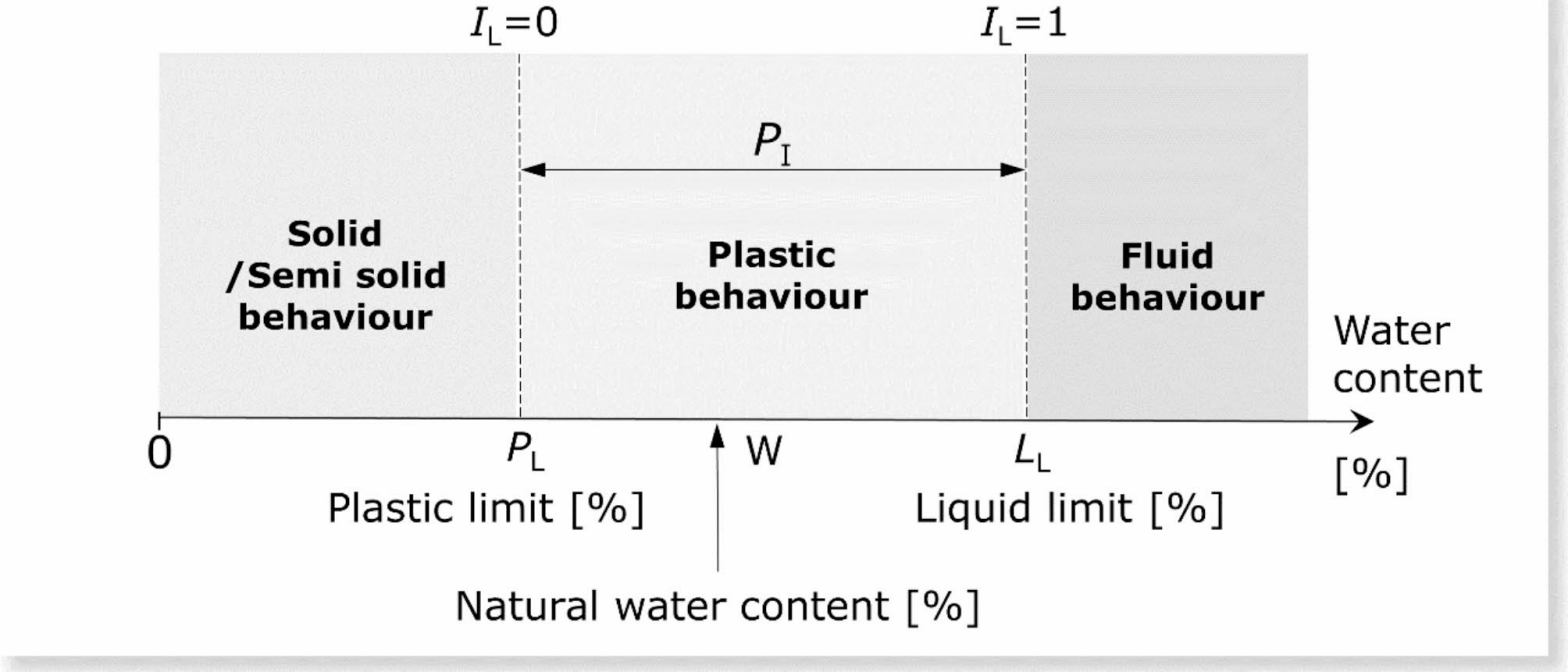
The graphical interpretation of the liquidity index. The figure enables the quantities pertaining to the sample’s water content, including natural water content, plasticity limit and liquidity limit, to be indicated on a collective axis. It demonstrates the range of soil moisture in different consistencies and correlates with the value of the liquidity index.

The graphical interpretation of Eq. (1) is shown in Fig. 1.

Macroscopic testing provides a very approximate assessment, often inaccurate for low-cohesive soils due to the low values of the plasticity index. For these soils, only a few percent difference in water content induces a change in the consistency. Laboratory testing of multiple samples is time-consuming and therefore, the ability to determine the *I*_L_ directly from in situ tests that provide a lot of data in a short timeframe is invaluable. The static sounding CPTU^3,4^ and dilatometer tests^5^ are the most commonly used in situ testing methods. The above-mentioned tests do not directly provide the *I*_L_ value, but the literature features various correlations for its determination, allowing these methods to be used in engineering practice to determine soil consistency, among other things. However, frequent errors resulting from the uncritical application of parameters on the basis of general interpretative Eq^6^ have been highlighted. Therefore, the implementation of reliable correlations derived for specific soils is crucial. This problem is particularly evident in the case of loess, for which it is difficult to find a formula that would allow a correct estimation of the liquidity index based on CPTU readings.

While the effect of water content on the properties of soils, particularly cohesive, is undeniable, a range of other determinants are also important. These include porosity, overconsolidation ratio, cementation, fraction distribution, degree of saturation, shape and grain alignment. The liquidity index *I*_L_ has become the commonly used leading parameter for the identification of geotechnical layers due to the fact that it can be determined without collecting intact soil samples and the use of sophisticated testing equipment, as well as the much shorter time of determination compared to strength and strain tests. However, the Polish commitment to the *I*_L_ indicator appears to be much higher than worldwide, which is possibly the result of the long-term application of the standard PN-B-03020^7^. According to the authors, a more appropriate and representative parameterisation of the subsoil, particularly for loess, is the use of the cone resistance *q*_c_ or the compressibility modulus M_DMT_. Nevertheless, *I*_L_ remains eligible to be used as an auxiliary parameter, mainly due to the fact that it allows for a prompt assessment of the quality of the subsoil. Considering the amount of data obtained through in situ tests, interpretive equations are therefore vital to derive valid geotechnical parameters from static sounding measurements, including, the determination of the *I*_L_ based on *q*_c_. These correlations ought to be calibrated for locally occurring soils taking into account, among other things, the grain composition, stress history and their genesis. The paper presents the results of the calibration of an interpretive formula for the determination of the liquidity index *I*_L_ based on the cone resistance *q*_c_ for eolian loess located in eastern Poland. The derived formula addresses the absence of suitable existing relationships for Lublin loess in the literature. It extends previous work described in^8^. The test and analysis methodology discussed in the paper is applicable for an arbitrary soil type for which the literature does not provide a reliable interpretive formula for estimating cohesive soil state based on CPTU tests.

### Loess subsoil

The research subject is the loess subsoil, which is a significant part of the earth’s land cover, extending approximately 6% of the global land area^9^. This special type of soil forming factors, as a rule, include wind. Good recognition of the properties of this soil is particularly important in areas where the loess cover is prevalent and serves as the primary foundation for construction. Examples of such areas include the Central Shandong Province (China), the Colorado Plateau (USA), the Sahel region (Burkina Faso) and the Lublin Upland (Poland). The mechanical properties of loess are strongly site-dependent, which is partly due to the type of accumulated material (eroded bedrock) and the specific conditions of its accumulation^10^. Experiences from studies on similar soils in other parts of the world can be taken as a reference point, but cannot be applied indiscriminately. Consequently, it is imperative to calibrate interpretive formulas to align with local conditions.

In geoengineering, aeolian processes are mainly identified with porosity and grain orientation, but the final properties of this subsoil were affected by other factors occurring simultaneously or subsequently, such as runoff, creep, sliding and water accumulation. Equally important is the extent of calcium carbonate cementation. Thus, the term ‘loess’ could be used to describe a wide variety of soils with complex genesis and diverse characteristics. Considering the classification of^10,11] and [12^, according to^13^, in geotechnical and geological-engineering studies, three main facies groups occur in the Lublin region: aeolian (typical loess), aeolian-diluvial and aeolian-alluvial.

**Fig. 2:**
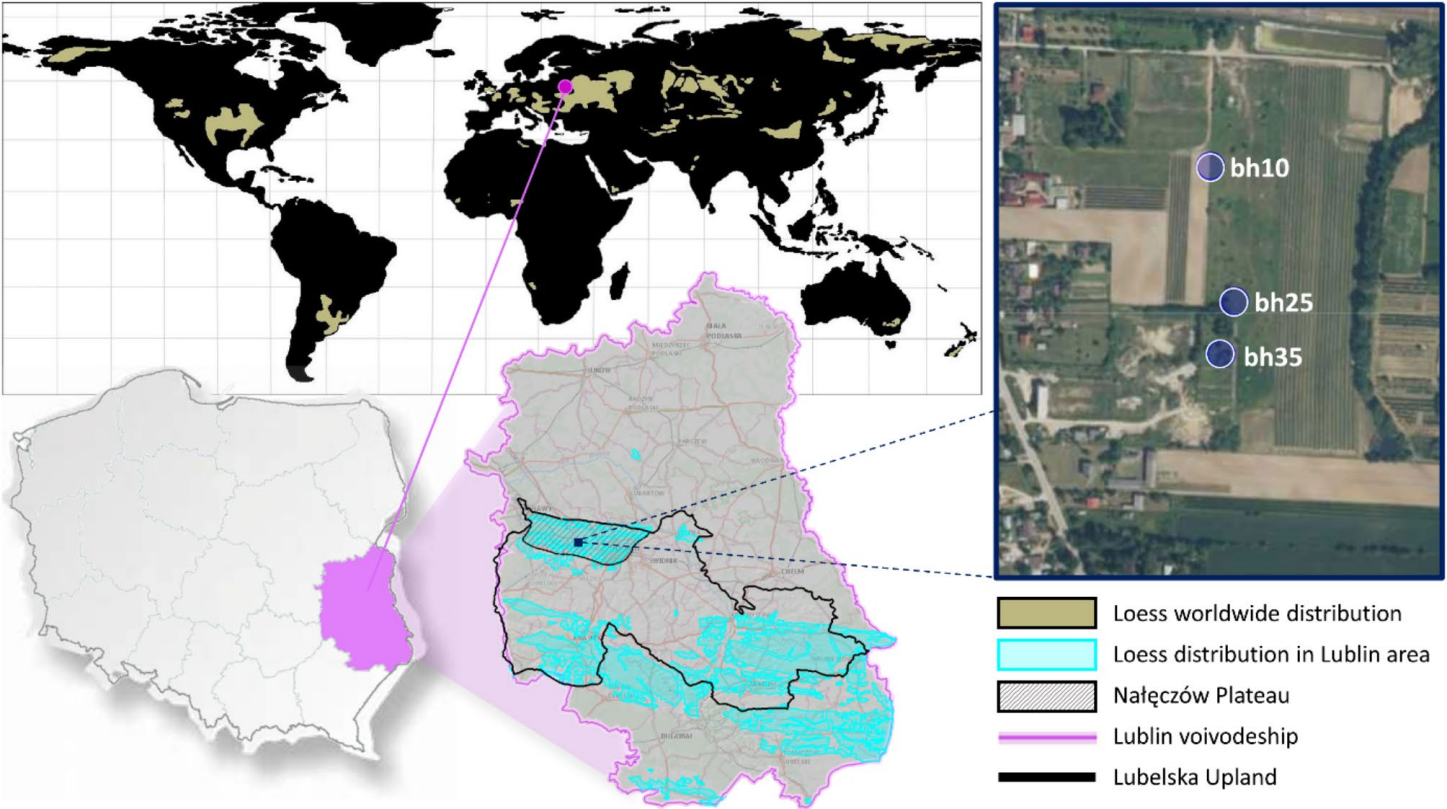
Research area location against worldwide loess distribution [the map base utilised in the figure was obtained from https://www.geoportal.gov.pl/]. The figure comprises areas at varying scales, which approximate the test plot from which the material for the study was derived. A simplified world map demonstrates the global distribution of loess deposits across all continents. The geographical position of Poland, within the context of the Lublin Voivodeship, is outlined, as is the topographical delineation of the Lublin Upland and the Naleczow Plateau. The test plot, together with the locations of boreholes 10, 25 and 35 from which samples were taken, are shown to the greatest approximation. A CPTU survey had previously been conducted at a distance of approximately 0.5–1.0 m from the sampling location.

Loess deposits occur mainly in southern and eastern Poland. One of the most well-known geographical regions of Poland identified with the prevalence of loess cover^14^ is the Nałęczów Plateau. The study area is located in the eastern part of Poland in the Lublin region and its natural boundaries are the Vistula River near Kazimierz Dolny in the west and the Bystrzyca River flowing through Lublin in the east. The study was conducted in a test plot in Piotrowice, located in the vicinity of Nałęczów, in the centre of the Nałęczów Plateau (Fig. 2).

The phenomenon inherently associated with loess is collapsibility^15–17^, when, as a result of pore saturation, intermolecular bonds are degraded, and rapid, significant settlement occurs. Loess soils susceptible to collapsibility are more porous, and in the Lublin region they are usually found in the near-surface zone, to a depth of about 3–4 m beneath the surface. The value of cone resistance *q*_c_ is related to both consistency (water content) and porosity. According to the study by Borowczyk and Frankowski^18^, collapsible loess is defined as that of aeolic facies in the solid state, with cone resistance *q*_c_<3.0 MPa. Therefore, reduced cone resistance will not always definitely indicate a change in consistency. Particular attention is required mainly in the near-surface zone. Furthermore, loess silt is a cohesive/non-cohesive transitional soil for which a small change in water content significantly affects the fluidity index, while its numerical evaluation by laboratory methods appears to be extremely problematic^16,19–22^. The amount of data obtained from laboratory tests is very limited compared to the capabilities of the CPTU. In addition, it is difficult to detect the limits of consistency change by macroscopic evaluation of the soil during borehole drilling, particularly in low-cohesive soils with smooth changes.

### Correlations between *I*_L_ and CPT results in the literature

**Fig. 3:**
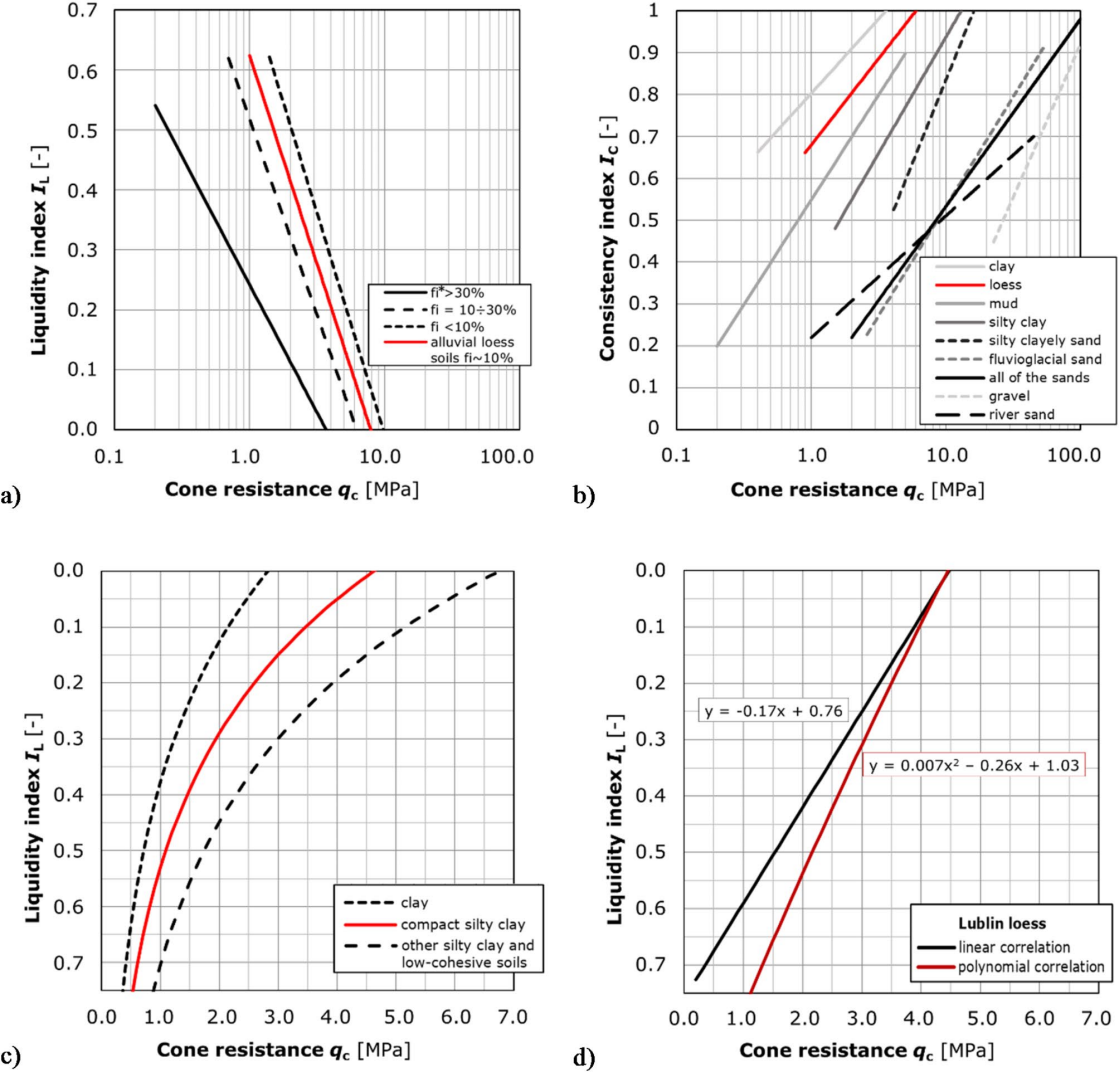
The interpretive formulae IL = f(qc) sourced from: (**a**) Polish Standard (PN-B 04452, 2002) and Frankowski, Pietrzykowski (Frankowski and Pietrzykowski, 2017); (**b**) ITB (Wysokiński and et al., 2008); (**c**) Wiłun (Wiłun, 1987); (**d**) Nepelski et al. (Nepelski, Lal and Franus, 2016); *fi – clay fraction content). A review of the related literature enabled the identification of correlations between the cone resistance values obtained from the CPTU test and the liquidity index across a range of soil types. This in turn facilitated the extraction of formulae that are declared to be specifically applicable to loess. The four nomograms presented in parts a, b and c from the selected and referenced studies enabled the applicability of the formulas to be verified for the loess of the study area, while the graph presented in part d represents the findings of the authors’ preliminary research conducted on loess samples from the Lublin region. The text provides a comprehensive analysis of the potential for estimating the liquidity index through the utilisation of diverse formulae, thereby establishing their accuracy.

The literature contains many attempts to derive the fluidity index from static sounding results. However, it is necessary to consider that, in view of the significant influence of various ancillary factors, without validation for the local soils, the correlations should be used with limited credibility. Of the multiple available formulae, selected and characterized were those which are possibly applicable to the analysis of the Lublin loess. The main nomograms are shown in Fig. 3. Equations that could be applied to the analysed soils are shown in red.

The correlation most frequently used in Poland is the one shown in the standard PN-B-04452:2002^23^, with three specific forms for the liquidity index, depending on the clay fraction content. The equations are intended for a range of values *I*_L_ ∈[0;0.7] and are not suitable for extrapolating results. Interpretation curve for *cohesive soils with the clay fraction content fi < 10%* shown in Fig. 3a and described by function:

2 $$\:{I}_{\text{L}}=0.729-0.736\text{log}{q}_{\text{c}},$$

should be appropriate for the Lublin loess, which is mainly classified granulometrically as silt.

The given expression was repeatedly analysed in terms of its suitability for the Lublin loess silts^8^, but the values obtained do not coincide with the results of laboratory tests. A considerably improved adjustment is obtained for the function designed for *soils with the clay fraction content fi in the range of 10–30%* described by the formula:

3 $$\:{I}_{\text{L}}=0.518-0.653\text{log}{q}_{\text{c}}$$

Reference^21^ suggested a modification of the above formulae. For alluvial loess from south-eastern Poland, they determined a new correlation, which is an interpolation between formulae (2) and (3).

Investigations carried out by Instytut Techniki Budowlanej^24^ on Polish soils led to the derivation of a set of regional relationships for loess. However, according to the authors, the established formula does not correspond perfectly to the Lublin loess and overestimates *I*_L_ in this case.

A relatively fair adjustment for the Lublin loess silts was shown by the interpretive path proposed by Wiłun (Fig. 2c)^25^, but in the author’s original intention it was meant for ‘*cohesive compacted clays*’. In contrast, the theoretically more appropriate formula, i.e. for *‘other clays and low cohesive soils*’, was found to have a lower degree of accuracy. According to Wiłun, the limiting values of *q*_c_ at which *I*_L_=0 are *q*_c_≈4.5 MPa for ‘*cohesive compacted clays*’ and *q*_c_ ≈ 7 MPa for ‘*other clays and low cohesive soils*’, respectively.

The loess silt deposits present in Lublin very frequently occur in a solid state, for which it is not possible to determine an exact *I*_L_ value using the above formulae^8^. developed their original preliminary correlation of *I*_L_ *=f(q*_c_*)* based on data obtained from static soundings mainly with the use of a mechanical cone (CPTM) and laboratory results. The relationships derived as linear and polynomial functions (Fig. 2d) are described by the following equations, respectively:

4 $$\:{I}_{\text{L}}=-0.17{q}_{\text{c}}+0.76$$

5 $$\:{I}_{\text{L}}=0.007{{q}_{\text{c}}}^{2}-0.26{q}_{\text{c}}+1.03$$

The given formulae showed a goodness of fit of R^2^ = 0.60 (for the linear function) and R^2^ = 0.61 (for the polynomial function). These relationships represented a pre-estimation and need to be confirmed in further research.

An interesting approach to the estimation of various geotechnical parameters was presented by^26^. Instead of submitting a direct interpretation path for the liquidity index, researchers applied the liquid limit and the plasticity index nomograms collectively. The results of their study, in a mathematically simplified form, are given by Croatian researchers^27^. According to their study, the liquid limit and the plasticity index are determined according to the following formulae:

6 $$\:{w}_{\text{L}}={10}^{(1.506+0.310\bullet\:\text{log}{F}_{\text{r}}-\text{log}{q}_{\text{t},1,\text{n}\text{e}\text{t}}/2.526)}$$

7 $$\:{I}_{\text{P}}={10}^{(\text{1,058}+\text{0,592}\bullet\:\text{log}{F}_{\text{r}}-\text{log}{q}_{\text{t},1,\text{n}\text{e}\text{t}}/2.206)}$$

Reference^22^ determined the relation between the liquidity index *I*_*L*_ and the net cone resistance *q*_*n*_, noting that excess pore water pressure can significantly affect the values adopted for correlation relationships. The formula developed applied to low-cohesive soils, among which the author included in particular highly sandy silts and clayey sands. He defined the correlation relationship between liquidity index and net cone resistance as:

8 $$\:{L}_{\text{L}}=a-b\text{ln}\left({q}_{\text{n}}\right)$$

where $\:{I}_{\text{L}}$ – liquidity index [–-], $\:{q}_{\text{n}}$ – net cone resistance, $\:a,\:b$ – regression coefficients.

For low-cohesive soils with a predominant silty fraction and values of the friction ratio *R*_f_ and normalized pore pressure ratio *B*_q_ specific for loess, the recommended values of the coefficients *a* and *b* are respectively 0.25 and 0.44.

In conclusion, despite the numerous relationships reported in the literature, most attempts to apply formulae directly according to granulometry usually result in deviations from the actual *I*_L_ values. Some formulae (e.g. Tschuschke or Wiłun) allow the estimation of *I*_L_ values close to those obtained in the laboratory, but this often requires the use of equations for soils with different granulometric characteristics and applies only to a narrow range of *q*_c_ values.

## Methods

**Fig. 4:**
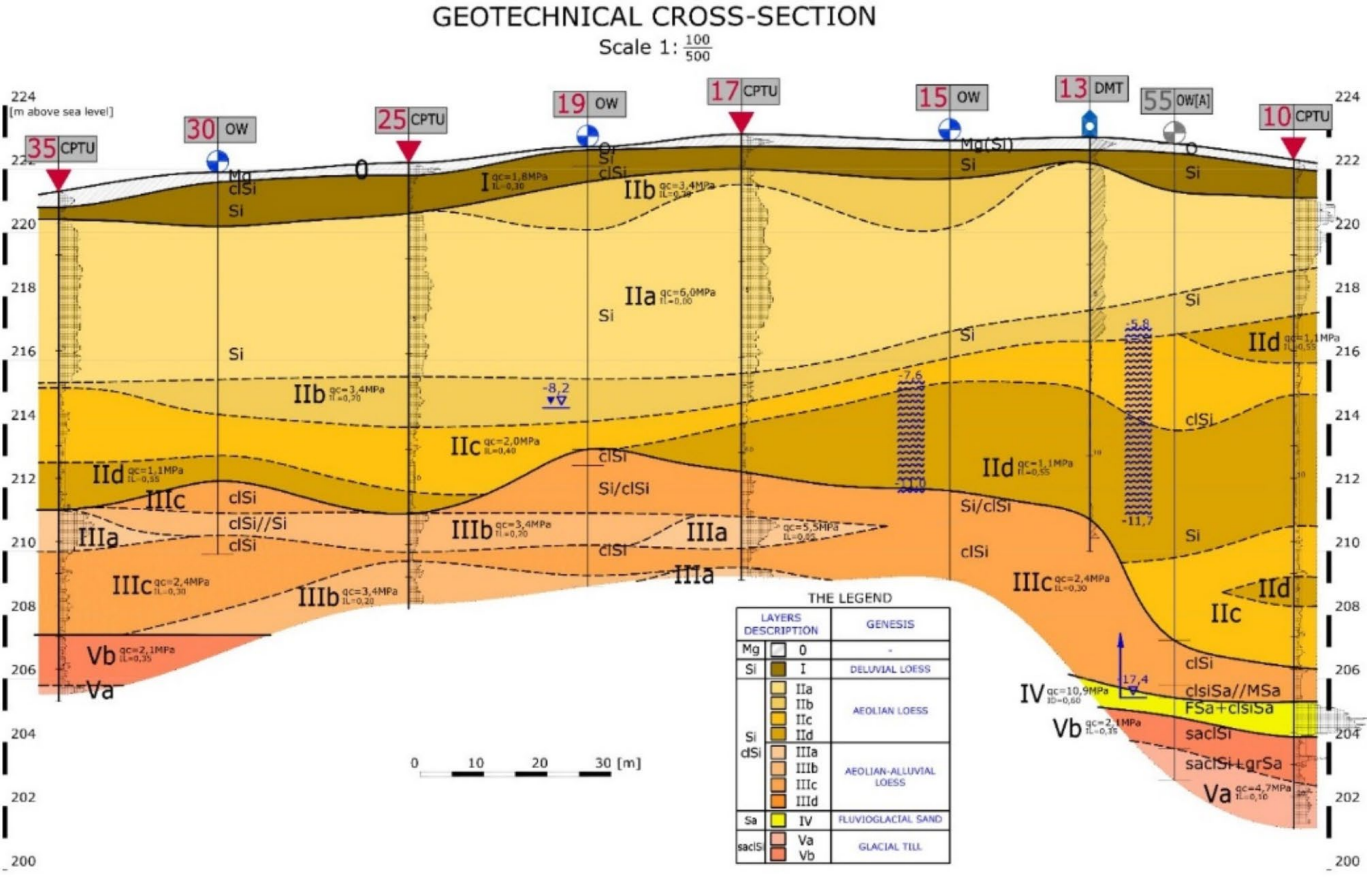
Cross section of the testing area. The detailed geotechnical cross-section provides a concise and informative representation of the subsoil within the study area. The boundaries between the separate geotechnical layers are illustrated, as are the names and symbols of the soils that comprise them. The location of test points, in the form of completed CPTU (including bh 10, 25 and 35, which are analysed in this paper), as well as additional in situ test locations and boreholes, are indicated. Furthermore, the cross-section supplies data regarding groundwater levels and the topography of the land.

The research was conducted in the vicinity of Nałęczów (Fig. 2), as part of an extensive geotechnical investigation, using in situ tests: CPTU, SDMT and PMT, as well as boreholes. The generalized subsoil structure of the studied area is shown in the cross-section (Fig. 4).

The covered loess of the aeolian facies, termed typical loess, is classified as silt and partly silty clay. In the Lublin region, these soils occur most frequently in a semi-solid to solid or hard-plastic state; however, a significant amount of the subsoil in the area concerned consists of plasticized soils. This prompted a calibration study that use static soundings.

Static soundings measurements were carried out with the use of the Pagani T63–200 penetrometer, which has a maximum pressure rating of 200 kN. The measuring cone was pressed at a rate of 2 cm/s, and data was recorded automatically at 1 cm intervals. The piezocone utilised in the present study was characterised by a standard geometry. The cone’s apex angle was 60 degrees, its area was 10 cm2, and the friction sleeve area was 150 cm2. The values measured included cone resistance *q*_c_, friction along the friction sleeve *f*_s_, and pore water pressure *u*. CPTU tests were carried out in accordance with the guidelines of ISO 22,476–1^28^.

Three representative test points (10, 25, 35) were selected to carry out CPTU static soundings, followed by boreholes in the immediate vicinity at a distance of approximately 0.5–1.0 m to collect samples for laboratory testing. Samples were obtained at 25-centimetre intervals during the dredging process of the borehole using a mechanical auger. This method corresponds to category B soil sampling, as defined by the EN ISO 22475-1 classification^29^. The soil sample quality class has been designated as 3 in accordance with Eurocode 7^30^ Following extraction, the soil was utilised to ascertain the natural water content and consistency limits. Each sample was tested in duplicate. In addition, the consistency limits were specified at approximately 1.0 m intervals. The plastic limit (*P*_L_) is assumed to be the water content at which the soil crumbles when rolled into a thread, in accordance with Atterberg’s definition. For each separated layer, approximately 7 g of rolls with visible cracks were taken into two pre-weighed crystallizing dishes. The material was then dried at a temperature of 105 °C (± 1 °C) for 24 h and subsequently reweighed to determine the water content corresponding to the desired plastic limit. The liquid limit (*L*_L_) was determined using the Casagrande method, which corresponds to the moisture content of the soil paste at which the groove made in the paste closes at a length of 1 cm and a height of 1 mm after the 25th drop of the cup on the base of the Casagrande apparatus. The *L*_L_ value was determined for each sample using at least six crystallizing dishes containing ground paste with varying water contents. For each moisture content, the number of drops, ranging from 12 to 38, required for the groove to close was recorded. These repetitions allowed for the plotting of the relationship between the number of strokes and the water content of the paste, thereby enabling the estimation of the soil liquid limit. The laboratory tests were conducted in accordance with ISO 17,892^31^, Part 1 (for natural water content) and Part 12 (for consistency limits).

**Fig. 5:**
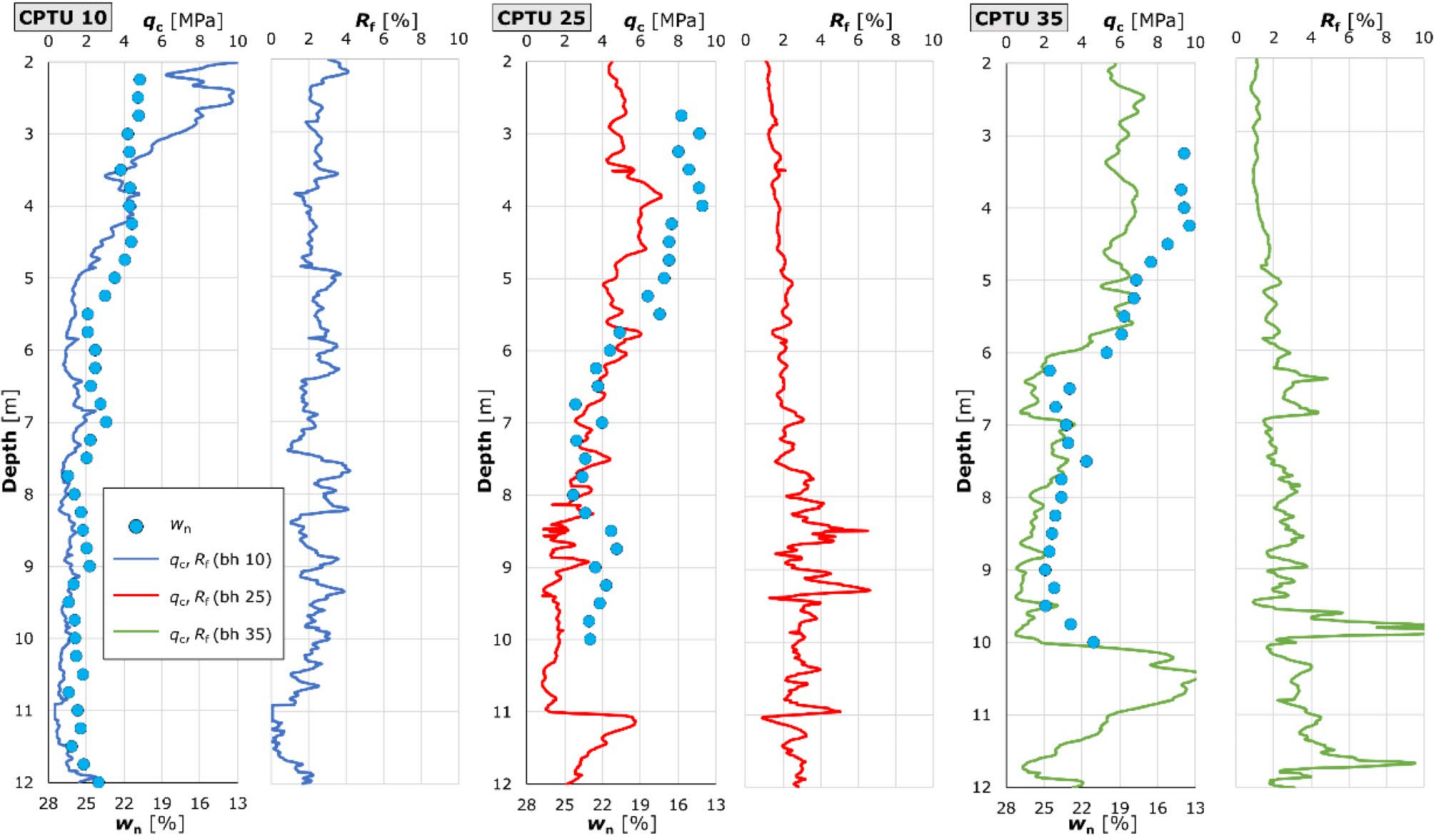
Soil water content w_n_ against cone resistance q_c_ and friction ratio R_f_ from CPTU. The figure illustrates the data obtained from the CPTU test, presented in the form of graphs of cone resistance and friction ratio for three selected tests, numbered 10, 25 and 35. For each test, the values of the natural water content of the soil samples, as determined in the laboratory, are plotted on the *q*_c_ graph. The samples collected comprised material from the layer, with a thickness of 25 cm, and their water content was determined as an average for the entire volume of the each sample. The graphs demonstrated a statistically significant association between the observed changes in moisture content and the corresponding values of cone resistance.

The sounding parameters, together with the natural water content established in the laboratory, are shown in Fig. 5.

### The characterisation of the studied soils

**Fig. 6:**
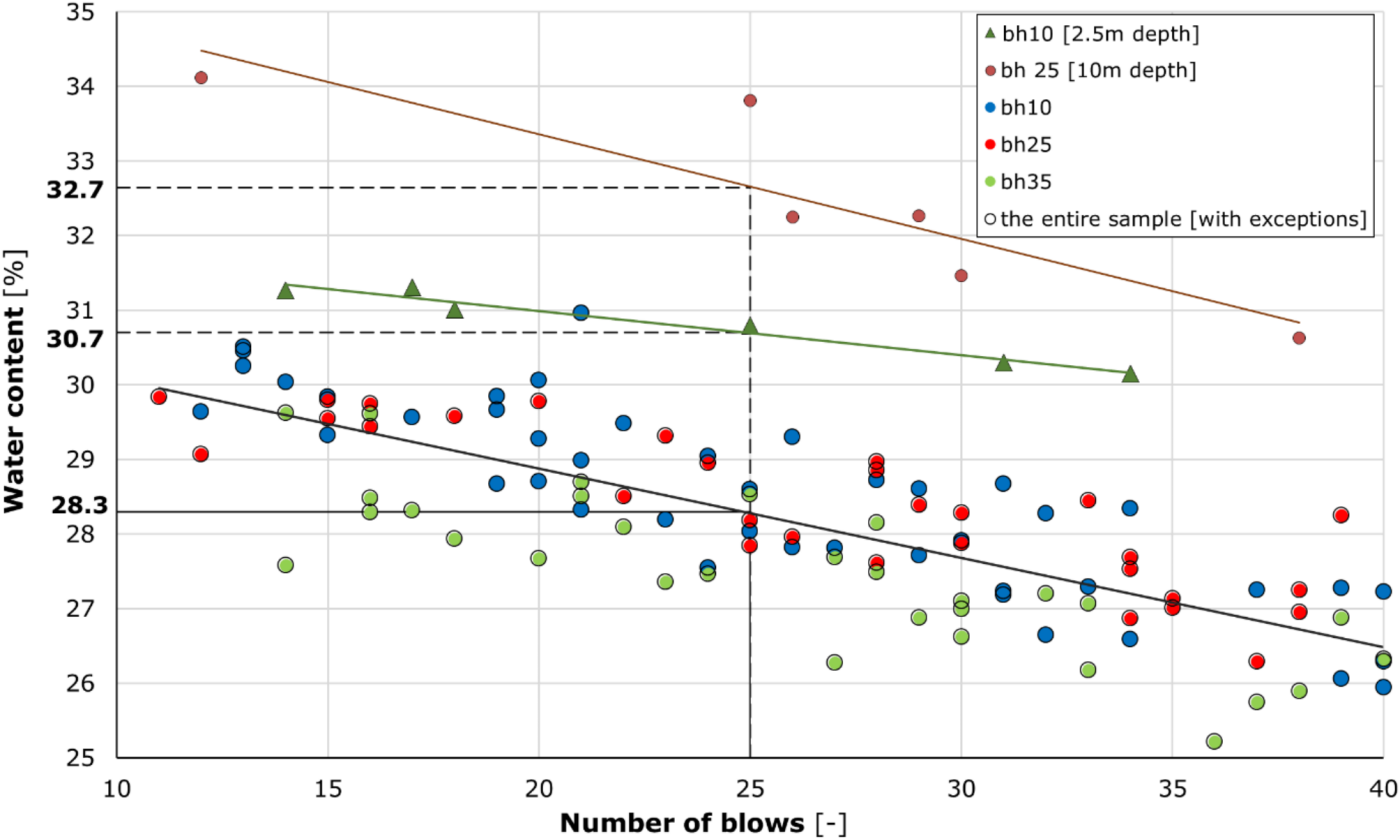
Summary of the results of the liquid limit (estimated with the use of Casagrande method). The chart represents a typical Casagrande liquid limit test. The coordinates of the points on the graph indicate the number of strokes of the apparatus bowl during the test for the specified soil paste, as a function of the moisture content. The results for the entire test sample are presented in a single graph, with the liquid limit determined separately for two samples with notably disparate characteristics. These include sample 10 from borehole 10, which was extracted at a depth of 2.5 m (*LL* = 30.7), and sample from borehole 25, which was extracted at a depth of 10 m (*LL* = 32.7). For all remaining samples, a single averaged liquid limit value of 28.3 was determined.

The natural water content for all of the tested samples ranges from 12.6 to 26.4%. In each of the boreholes, lower water content occurs near the surface down to approximately 4.5–6.0 m and increases with depth. In boreholes bh25 and bh35, near-surface water content fluctuates between 12 and 16%, and then gradually increases below a depth of about 4.5 m, reaching values of 21–25% below a depth of 6.0 m. In borehole bh10, silt with a water content of more than 20% is found below a depth of 2.0 m, while below about 5.5 m, there is a marked increase in water content to approximately 26% (± 0.5%). Liquid limits range from 26.8 to 32.7%. Except for two samples, bh10 [2.5 m depth] (*L*_L_ = 30.7%) and bh25 [10 m depth] (*L*_L_ = 32.7%), the others are marked by very similar results. Liquid limits of 28%±1% were obtained from both the individual test and from the summary plot of all the results as shown in Fig. 6. This proves the high lithological homogeneity of the subsoil in the investigated area. This also applies to the plastic limits, which show similar variability. Apart from sample bh25 [10 m depth] (*P*_L_ = 18.4%), the plastic limit amounts to 20%±1%.

The plasticity index *I*_P_ of the majority of the samples varies between 6.5 and 9.7%, which classifies the soils as low cohesive (silt) according to the standard^32^. Only the results for samples bh10 [2.5 m depth] and bh25 [10 m depth] classify them as medium cohesive with the plasticity index *I*_P_ =10.5 and *I*_P_ = 14.2%, respectively.

**Fig. 7:**
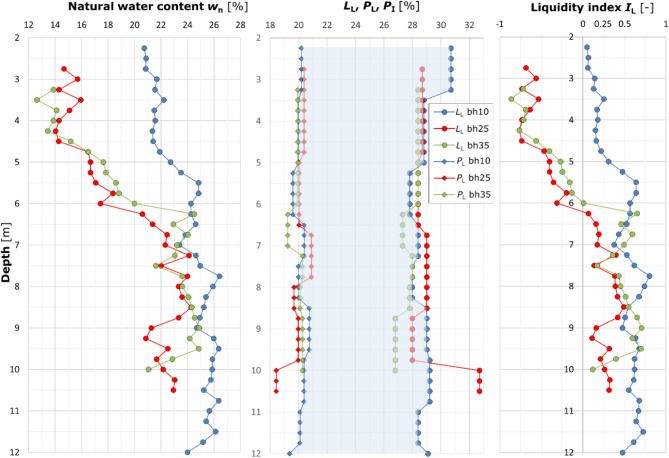
Summary of laboratory results. Distribution at depth: natural water content; Atterberg limits; plasticity index. The three presented graphs illustrate the laboratory results obtained for the collected loess samples. The first graph illustrates the natural water content, the second depicts the consistency limits and resulting plasticity index, while the third graph indicates the liquidity index values. The graphs illustrate the variation in the determined quantities at depth for each borehole.

The completed analyses allowed the development of a quasi-continuous subsoil profile parameterized by natural water content, Atterberg limits, plasticity index and fluidity index. Figure 7 shows the summarized laboratory results for all samples collected.

**Fig. 8:**
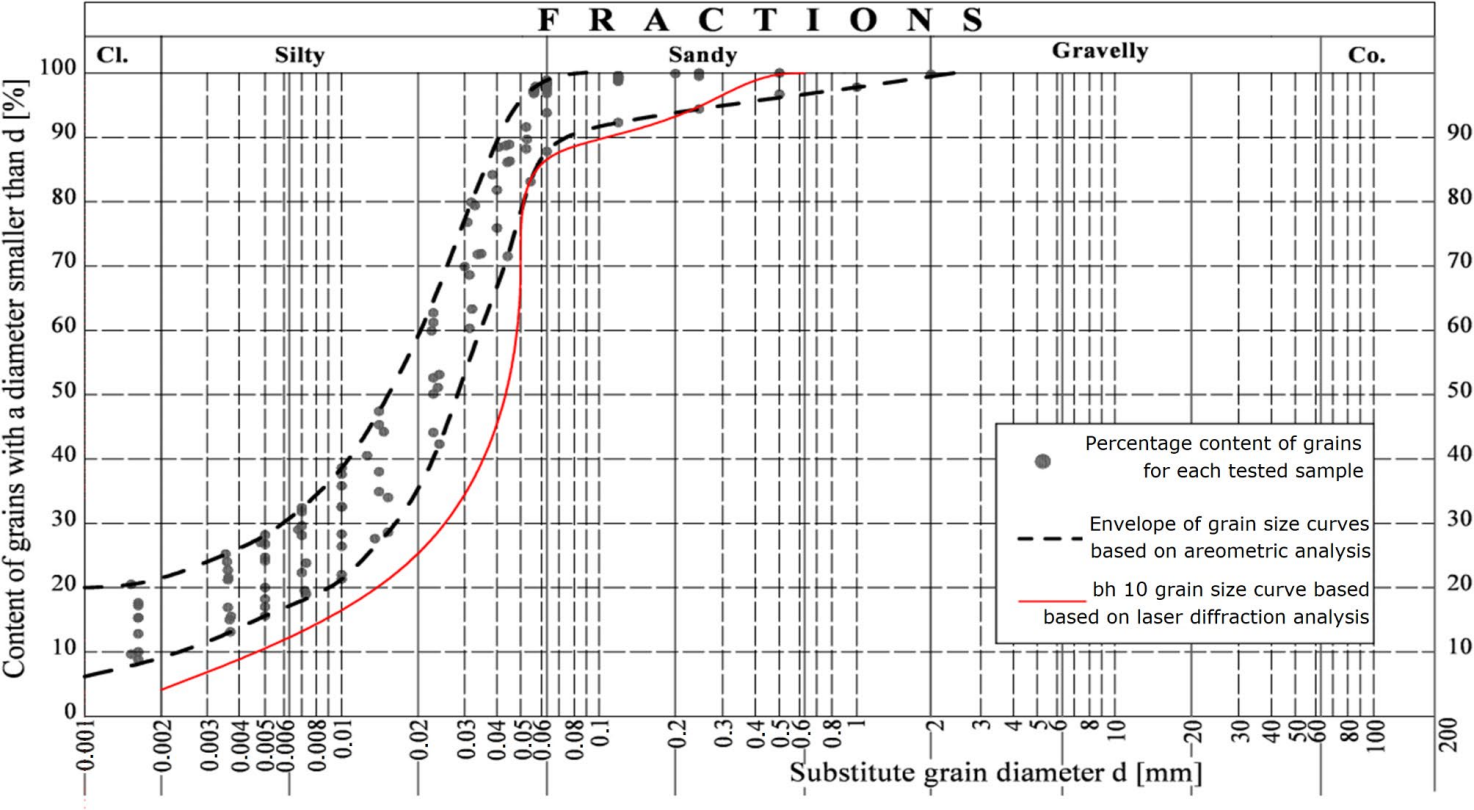
Grain size distribution bh10, bh25, bh35. A crucial element of soil laboratory testing is the identification of its grain size. Loess from the study area underwent areometric analysis, with select samples also subjected to laser diffraction analysis. The conducted tests resulted in the establishment of limiting grain size curves for the soil, defining the maximum content of individual fractions.

For representative samples, granulometric analyses were also performed using the areometric method according to^33^. In addition, for the selected sample bh10, a laser raction study was conducted with the use of a particle size analyser. The data obtained enabled the derivation of limit grain size curves for the soil from the research field shown in Fig. 8.

In summary, the investigations reveal that the soil states range from solid to hard plastic down to a depth of about 5–6 m below the surface. In the deeper parts of the subsoil, a significant increase in plasticity is observed with depth. It should be highlighted that the trend of the *I*_L_ values from the laboratory tests coincides with the resistance of the cone *q*_c_.

**Fig. 9:**
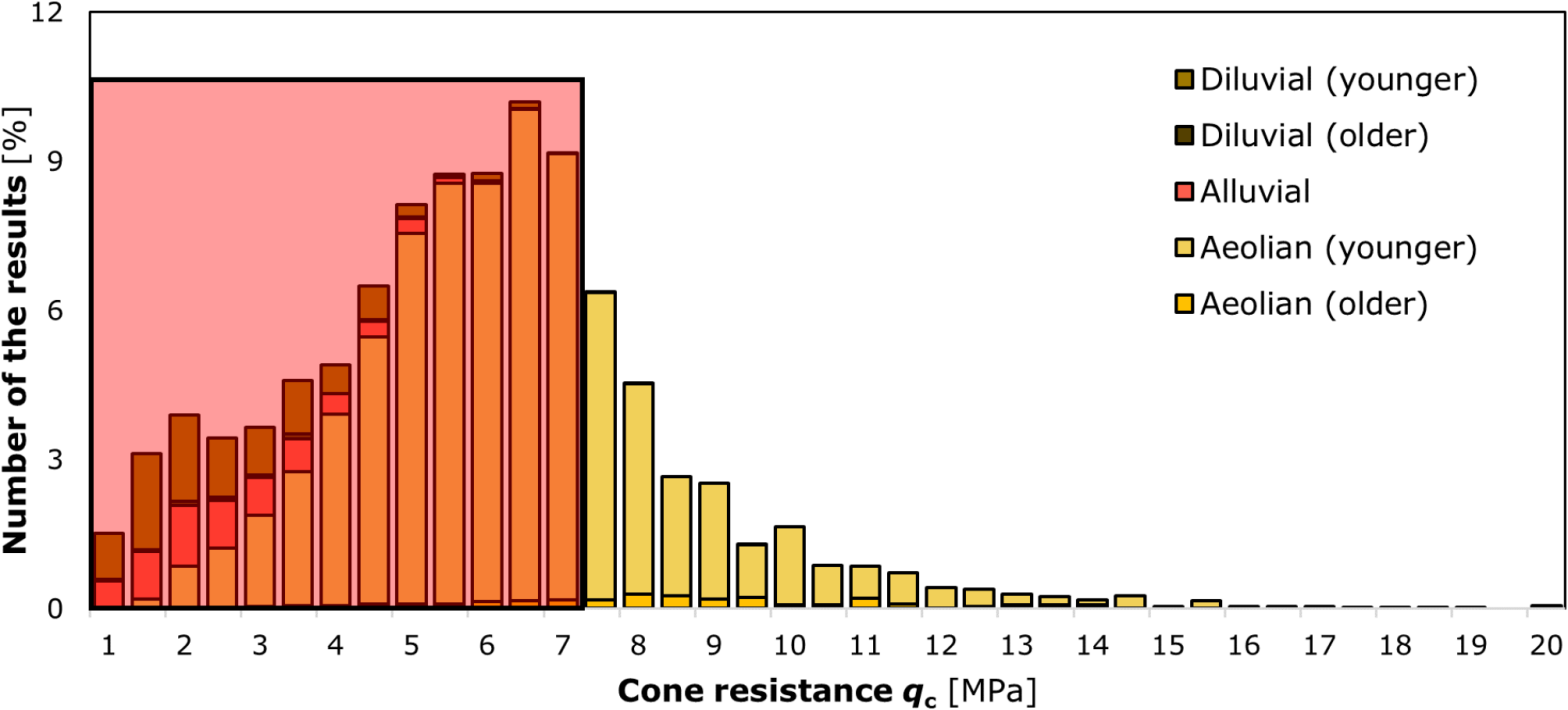
Distribution of qC values from CPT static soundings in the Lublin area with division into facies. The diverse morphological characteristics of a given soil type are indicative of the specific climatic conditions under which they were formed. Typically, aeolian loess were formed through the wind erosion of the bedrock. Additionally, these materials were transported by wind and deposited under arid conditions, exhibiting minimal alteration from their original characteristics throughout the sedimentary process. Meanwhile, deluvial and alluvial loess were subjected to other processes, resulting in the evolution of formations with disparate characteristics from those of typical loess. As a result of the extensive research conducted previously by the authors, a statistical distribution of the characteristic *q*_c_ values for the individual facies was established. The results obtained for loess from the research plot are marked by the red square in the diagram, which serves to confirm the representativeness of the research sample for loess in the Lublin region.

Cone resistances *q*_c_ ranged from 0.6 to 7.7 MPa (95% of the results). With reference to the distribution of these values registered in the Lublin area^34^ (Fig. 9), which is comparable to the loess of the Nałęczów Plateau, the soils investigated were located in its lower part.

**Fig. 10:**
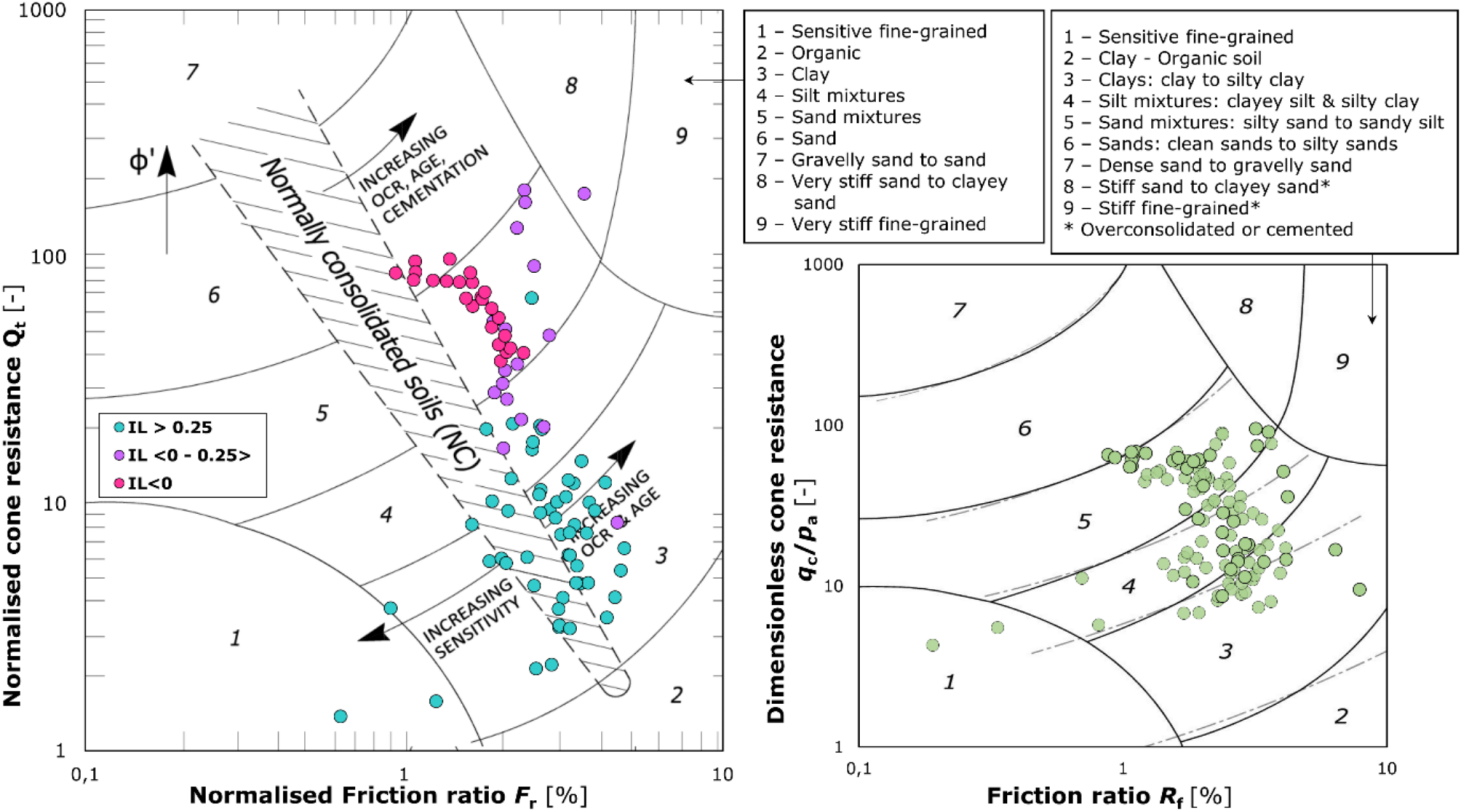
CPT data against the SBT nomograms^35,36^. The soil behaviour charts developed by Robertson in 2010 and 2016 facilitate an initial classification of the soils under investigation based on their cone resistance and friction ratio in CPTU in situ test. These quantities are then expressed as normalised values or plotted on the nomogram based on the dimensionless direct results. The indications provided by the nomograms serve only as a preliminary estimate of the soil type, which is then confirmed by the results of test boreholes with macroscopic analysis and additional laboratory tests performed on representative samples.

Inherent in the interpretation of static soundings is the determination of the soil type using classification nomograms. The identification makes it possible to the specify the soil type, which, however, cannot be strictly related to the granulometric composition, as it is an indication of soil behaviour, the so-called SBT (Soil Behaviour Type). Therefore, there are occasionally discrepancies between the results of recognition using nomograms and actual soil composition. The sounding data were plotted on the two most popular Robertson nomograms (Fig. 10), dated 2016^35^ and 2010^36^. Both nomograms indicate behaviour of the examined samples within silty soils.

## Results

**Fig. 11:**
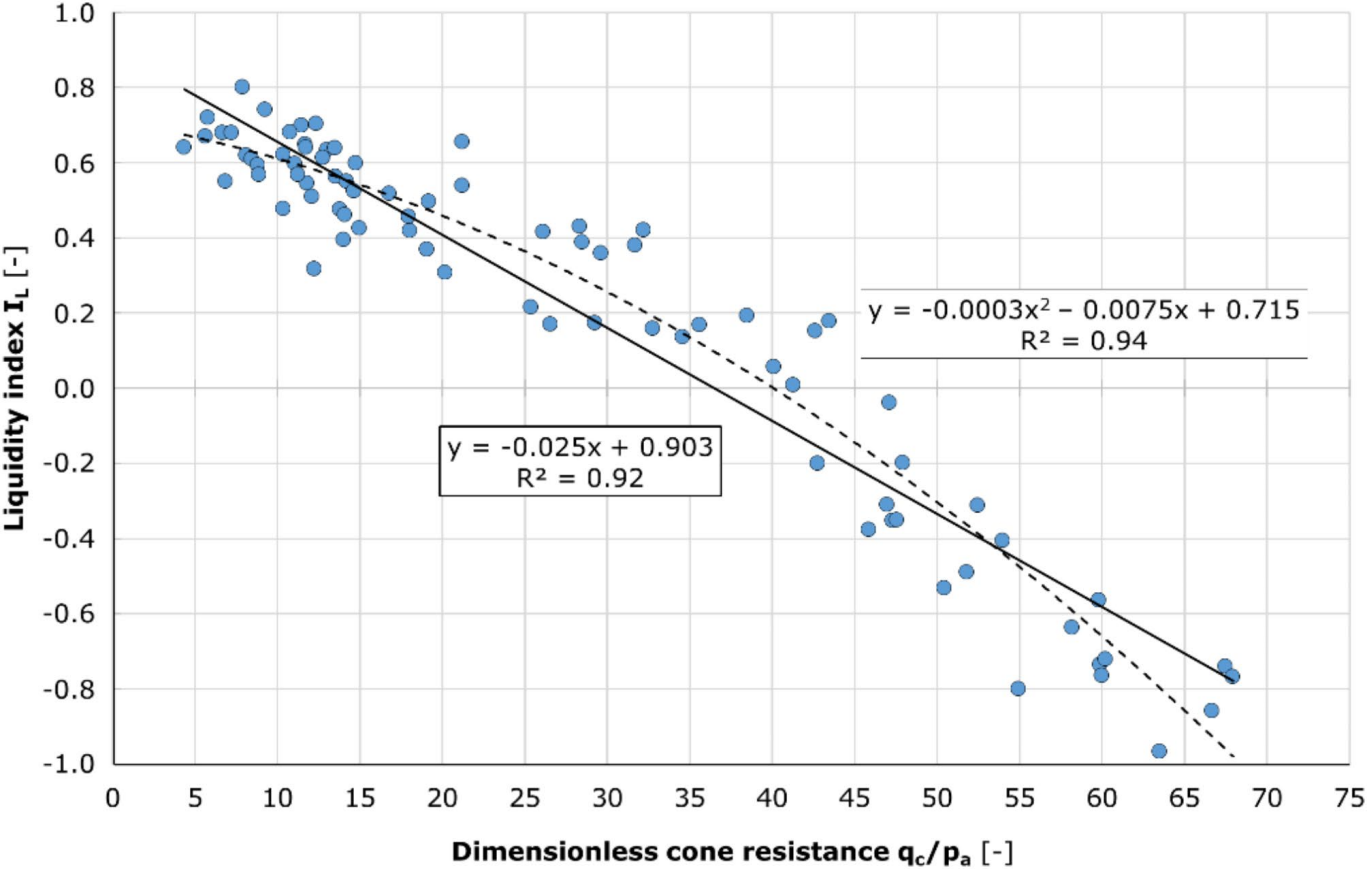
Liquidity index as a function of the dimensionless cone resistance. A detailed examination of the laboratory-based liquidity index results and the corresponding average cone resistance values enabled the formulation of a linear and polynomial function that accurately represents the observed relationship. Furthermore, the figure illustrates the coefficient of determination (R^2^) for each function, providing evidence of the function’s reliability in aligning with the empirical outcomes.

The primary aim of the research described was to determine the correlation function between the cone resistance *q*_c_ and the liquidity index *I*_L_. For this purpose, the *I*_L_ values obtained from the laboratory tests were compared with the cone resistance *q*_c_. The *q*_c_ value was taken as the average of the readings over the ± 25 cm depth range corresponding to the sample taken. The (*q*_c_/p_a_) – *I*_L_ pairs were then plotted in Fig. 11.

Results significantly deviating from the trend were excluded from further analysis. The remaining data indicated a covariance of -8.77, with a Pearson coefficient of -0.96. The following linear and polynomial relationships were derived:

9 $$\:{I}_{\text{L}}=-0.025\bullet\:{(q}_{\text{c}}/{p}_{a})+0.903[-]$$

10 $$\:{I}_{\text{L}}=-0.0003\bullet\:{({q}_{c}/{p}_{a})}^{2}-0.0075\bullet\:{(q}_{\text{c}}/{p}_{a})+0.715[-]$$

in which cone resistance *q*_c_ is given in MPa.

For the polynomial function (10) a goodness of fit of R^2^ = 94% was obtained, while for the linear regression (9) a slightly lower R^2^ = 92% was found.

Furthermore, in order to exclude the effect of test depth on the cone resistance value, the IL variation was analysed against the normalised cone resistance *Q*_t_, where:

11 $$\:{Q}_{\text{t}}={(q}_{\text{t}}-{{\upsigma\:}}_{v0})/{{\upsigma\:}{\prime\:}}_{v0}$$

**Fig. 12:**
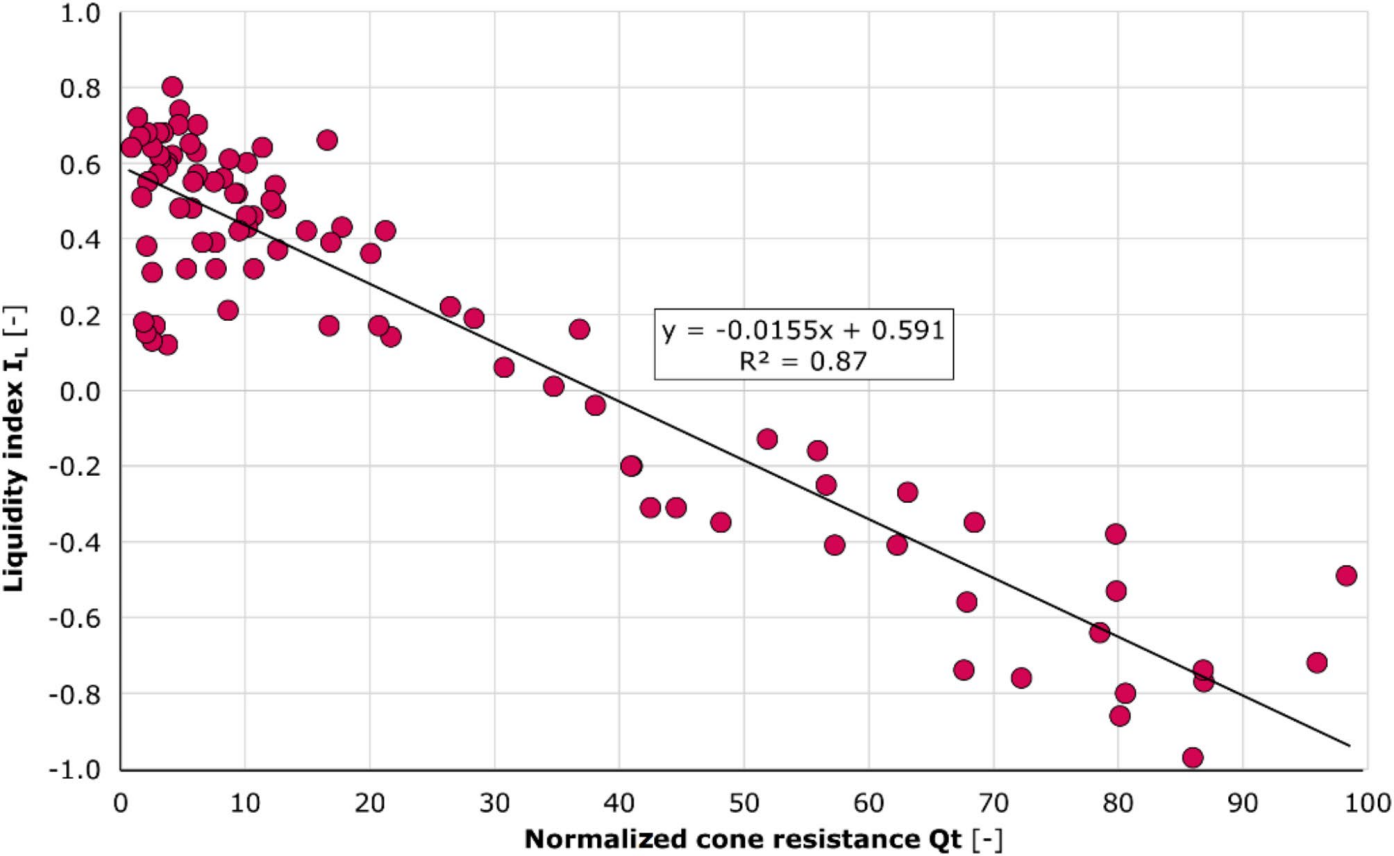
Liquidity index as a function of the normalised cone resistance Qt. Further analysis involved the examination of liquidity index results and the corresponding average normalised cone resistance, as illustrated in the diagram. A linear relationship was identified. Additionally, the figure indicates the coefficient of determination (R^2^) for established function, which provides evidence of the reliability of the formula’s fit with the empirical results.

Similarly to the previously established relationship between the *I*_L_ and *q*_c_/*p*_a_ values, a significant correlation formula was identified between the quantities under consideration. The calculated covariance were − 11.49, while Pearson’s coefficient − 0.94. Furthermore, the linear correlation formula yielded a coefficient of determination R^2^ of 0.87, indicating a strong relationship between the variables, as shown in Fig. 12.

Finally, a linear regression formula (9) binding *I*_L_ and *q*_c_/*p*_a_ values was adopted for further analysis. This was decided because of the small difference in accuracy, while simplifying the calculation of the liquidity index in practical use. The regression constant obtained in the results evaluation indicates that the maximum possible *I*_L_ value is 0.903, while *q*_c_ is equal to 0. Although it is generally possible to obtain a soil moisture level that exceeds the plasticity index, it is very unlikely that such an occurrence would be observed in the subsoil. Furthermore, during the penetration of the piezocone into the subsoil, the value of the resistance *q*_c_ is never equal to 0. In the entire test sample analyzed, the maximum laboratory-determined value of *I*_L_ is 0.8, while the lowest single recorded cone resistance is 0.35. Consequently, the regression constant appears to be a highly probable value, suggesting that the test sample has been adequately analyzed. Furthermore, with use of the linear regression (9), it is possible to determine the limiting values of cone resistance *q*_c_ for individual consistencies^31^, which are summarized in Table 1.

**Table 1:** The limiting values of cone resistance *q*_c_ for individual consistencies.

Consistenty stage	Liquid	Plastic				Semi-solids	Solid
Description	Liquid	Very soft	Soft	Medium soft	Stiff	Very stiff to hard	Hard to very hard
I_L_ [–]	> 1	1–0.75	0.75–0.5	0.5–0.25	0.25–0	< 0	< 0
q_c_ [MPa]	< 0.62		0.63–1.62	2.63–1.63	2.63–3.64	> 3.64	

**Fig. 13:**
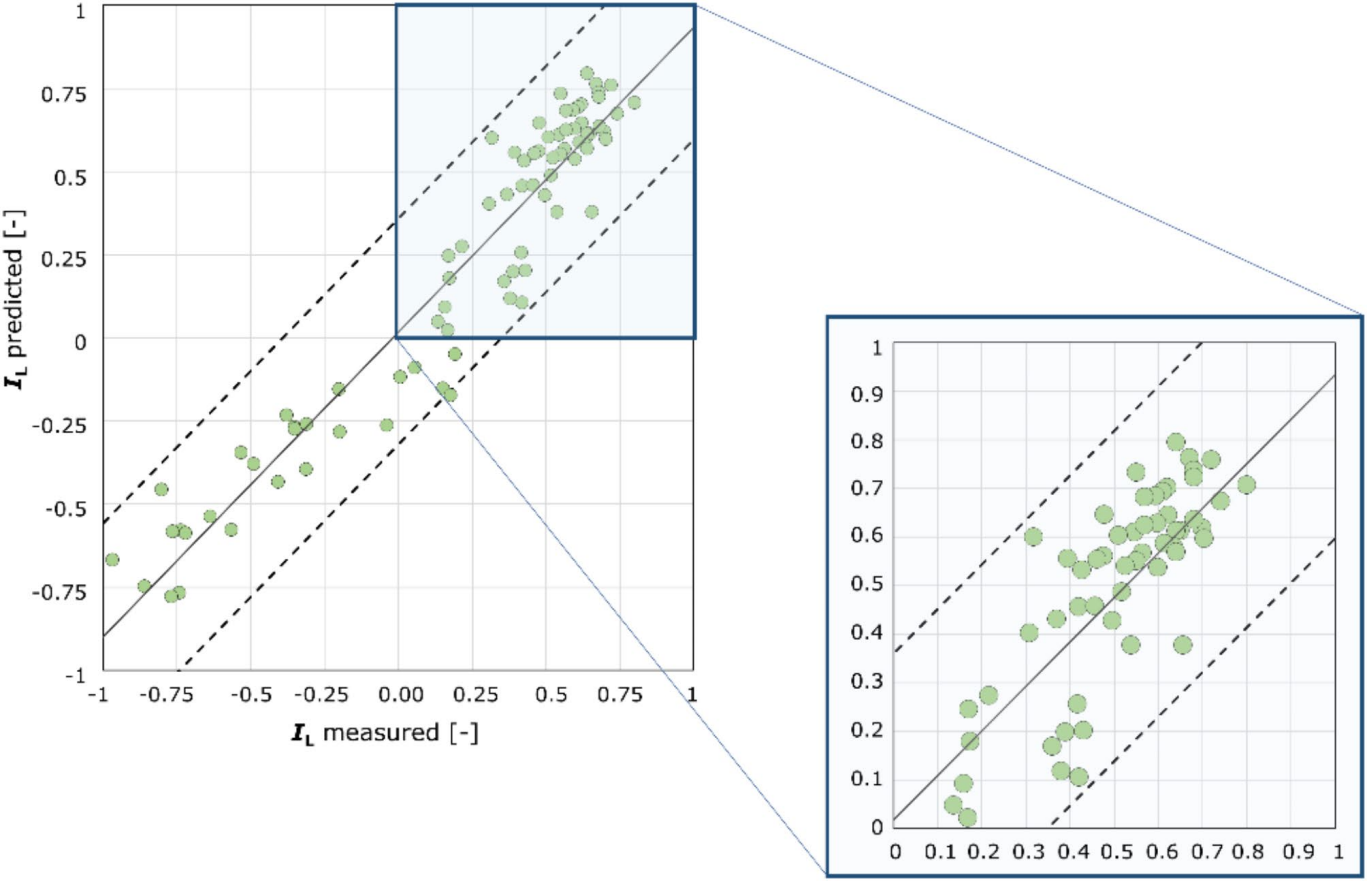
Comparison of I_L_ values interpreted with the new formula. The figure provides a graphical representation of the relationship between the liquidity index values obtained from the tests and the IL values estimated from the linear function derived from the tests outlined in the paper. Furthermore, the graph illustrates the maximum range of results, presented on a larger scale.

To validate the derived formula, Fig. 13 compares the *I*_L_ values from laboratory tests with those obtained from Eq. (9). The solid line shows the averaged trend, while the dashed lines show the range of *I*_L_ values ± 0.3.

The specified range of results allows for the verification of limiting cone resistance in relation to different soil consistencies and states. In the analyzed subsoil, semi-solid and solid consistencies were observed when the cone penetration value exceeded 4.4. Stiff soil was predicted for *q*_c_ values ranging from 3.25 to 4.4, while medium-soft soil was expected for values between 1.72 and 3.25. Soft soil was identified with *q*_c_ values ranging from 0.8 to 1.72. Cone resistance *q*_c_ values obtained from CPTU tests lower than 0.8 indicate the presence of very soft soil. The verified limit values of qc should be regarded as reliable for assessing the quality of the aeolian loess subsoil in eastern Poland for construction, based on CPTU test results. This reliability is attributed to the fact that higher water content in cohesive soil complicates the conditions for the foundation of engineering structures.

**Fig. 14:**
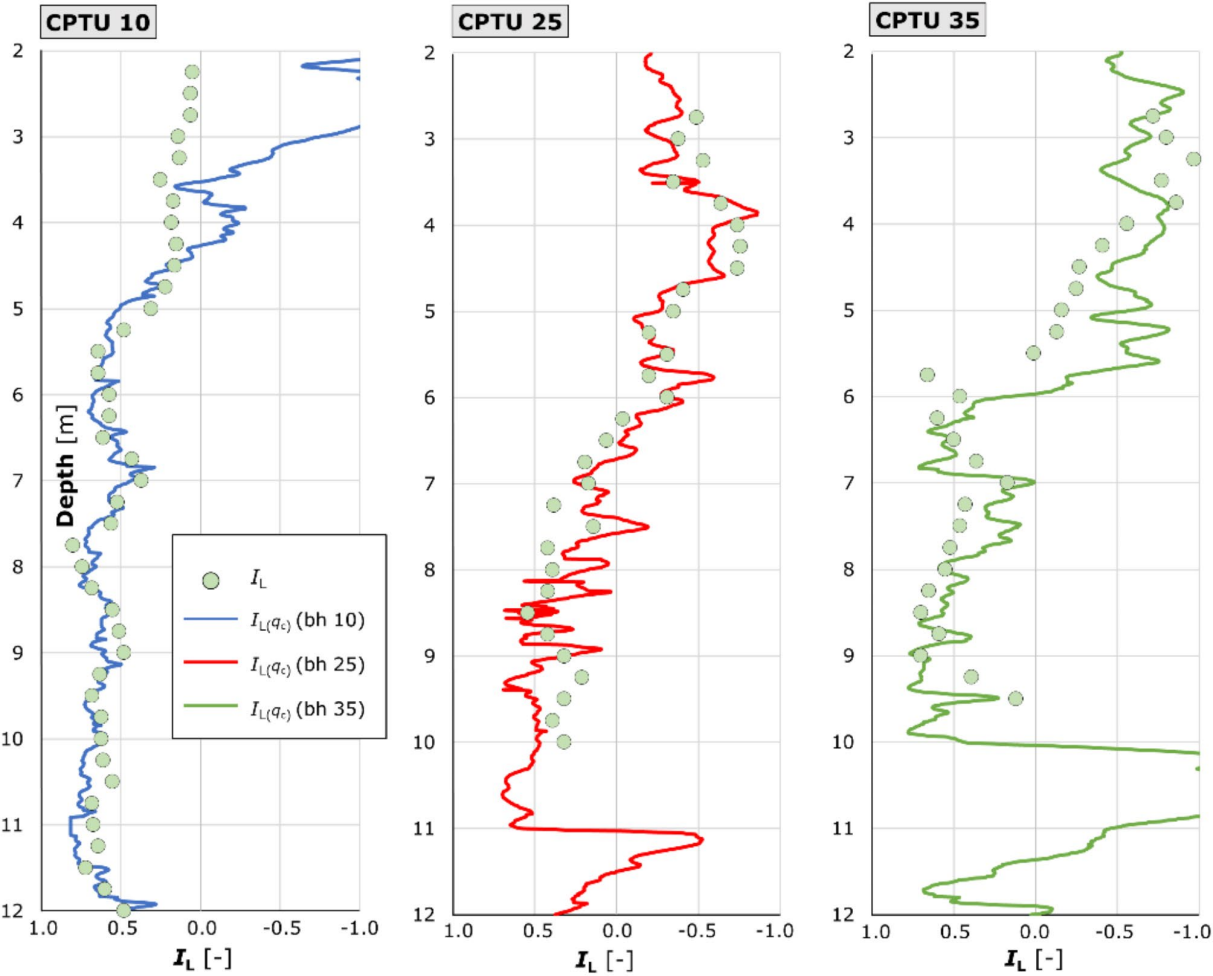
is a summary of the laboratory *I*_L_ values against the continuous profile estimated from Eq. (9) in each borehole.

Figure 14 Comparison of laboratory I_L_ values with results obtained using the estimated formula based on q_c_. The graphs illustrate the point results of the laboratory liquidity index determined on the basis of samples from boreholes 10, 25 and 35. Additionally, they reflect the continuous at-depth *I*_L_ values obtained through the interpretation of the *q*_c_/*p*_a_ quantities. The results presented in summary form facilitate comparison between the calculated values and the laboratory-determined values.

**Fig. 15:**
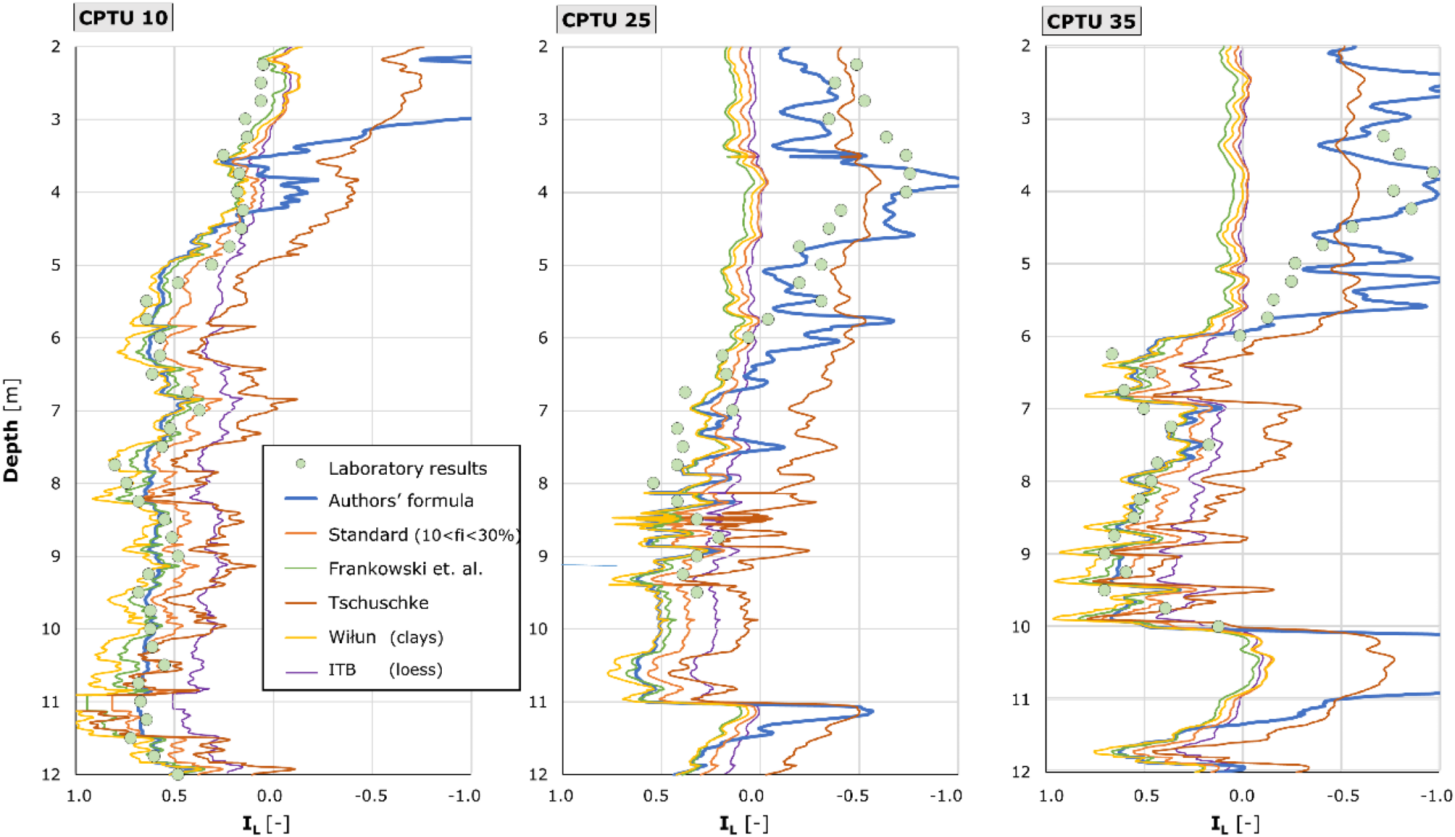
Summary of interpretation equations with the highest alignment. The diagrams prepared for the three test boreholes demonstrate a variation in *I*_L_ values with depth. These values have been estimated on the basis of interpretation formulae that have been accepted as appropriate for the analysis of Lublin loess in the context of published literature. The Liquidity Index was calculated in accordance with the formula developed by the Polish Standard for cohesive soils containing a clay fraction of between 10 and 30%, as proposed by Frankowski, Tschuschke, and Wiłun (for clays) and as outlined by the ITB. Furthermore, the IL values derived from the authors’ research methodology are indicated.

A comparative evaluation of the *I*_L_ values determined by the selected formulae from the literature that showed the greatest compatibility with the laboratory test results was prepared for the synthesis. In addition to the Eq. (9) determined on the basis of the research performed, formulae established in the Standard^23^, according to^21,22,24,25^ were adopted for comparison. In the case of the Tschuschke equation, the normalized cone resistance *q*_n_ was used to calculate the *I*_L_ value. A summary is shown in Fig. 15.

**Fig. 16:**
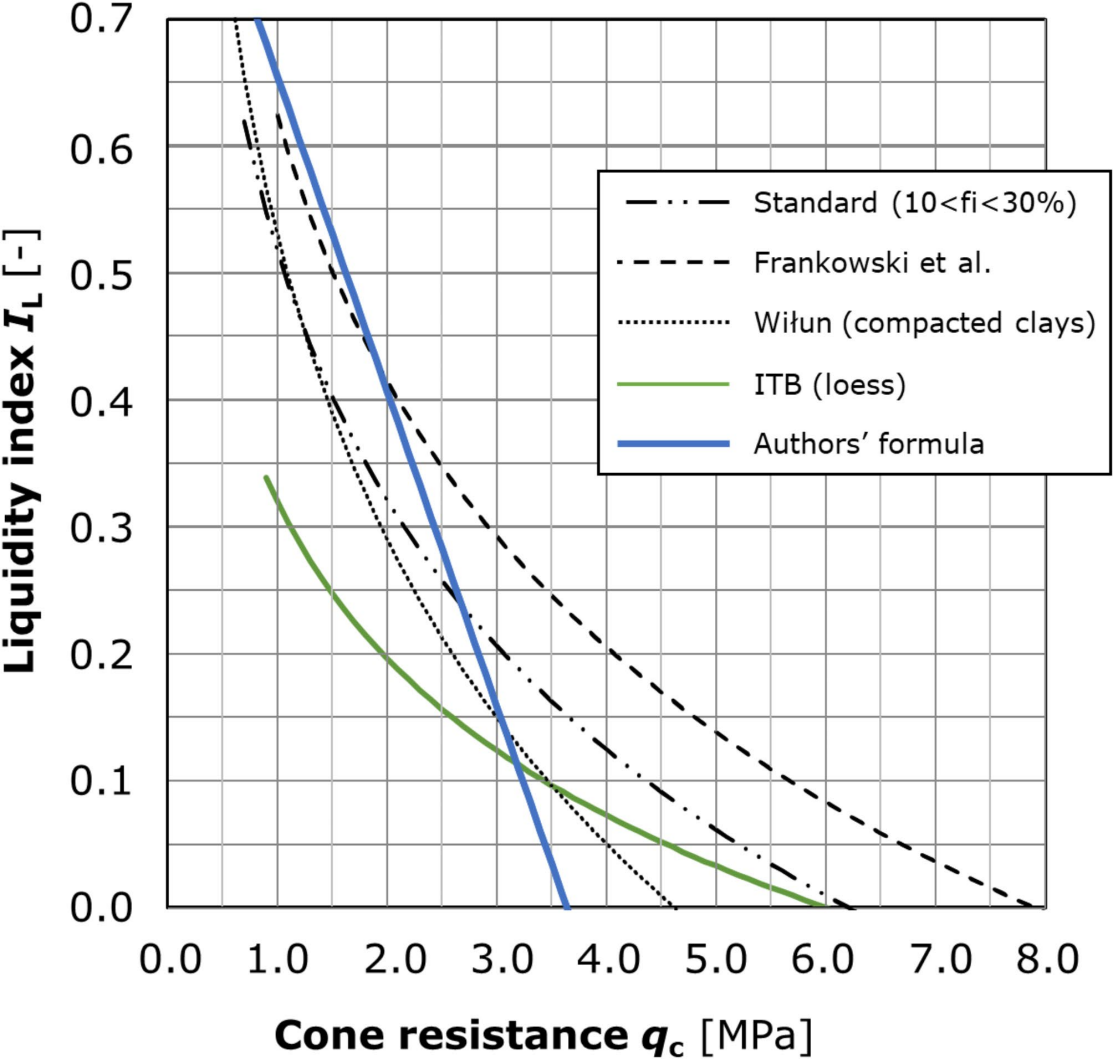
The correlation applicable to the Lublin loess I_L_ = f(q_c_) sourced from: the Polish standard^21,23^; ITB (Wysokiński et al., 2008);^25^ and present research. The nomogram offers a synthesis of available functions, which, when applied to a known *q*_c_ value expressed in MPa, enables the estimation of the predicted liquidity index for the Lublin loess. The nomogram serves as a valuable engineering instrument for the expeditious estimation of subsoil quality at the initial stage of in situ testing.

For loess occurring in the Lublin region, the authors suggest the use of a linear relationship established on the basis of calibration tests described in this article. In addition to the use of the formula (9), it is also possible to determine the *I*_L_ based on Fig. 16.

The nomogram includes both, a linear function developed by the authors and the collective literature formulae applicable for loess. Despite the very good convergence obtained in the tests performed, the authors consider that the *I*_L_ value should be an auxiliary parameter, while the *q*_c_ should be the leading value for the identification of geotechnical layers. However, subsoil identification cannot be based on soundings solely and the selected tests should be verified by boreholes. It is essential to carry out boreholes and soundings at nodes to improve interpretation. In practice, loess often shows reduced *q*_c_ values that do not correlate with a change in state. This is mainly the case in the near-surface zone, usually to a depth of 3–4 m. This is explained by the fact that the *I*_L_ value depends mainly on the natural water content and clay fraction content, but *q*_c_ is also influenced by a number of other factors, e.g. porosity, cementation, in-situ stresses and overconsolidation ratio. In the case of loess, reduced cone resistance may be related not only to the condition but also to the structure of the soil and be an indicator of its collapsibility.

In instances where high porosity of the soil is present in a solid or semi-solid state, the observed *q*_c_ values are notably lower. It has been estimated that in cases where *q*_c_ values are less than 3 MPa^37^, in conjunction with *I*_L_$\:\approx\:$0 and reduced cementation, loess is more susceptible to collapsibility. This phenomenon is particularly pronounced in the presence of macropores within the soil structure. Furthermore, higher *q*_c_ values have been shown to be indicative of a higher pre-consolidation index^38^ and increased cementation of soil particles.

The derived formula is considered to be a reasonable adaptation for plasticized loess, whereas at low water content, the importance of other factors increases. It is therefore recommended to use the derived formula (9) for the determination of *I*_L_ in the range 0.0 ÷ 1.0.

Furthermore, an attempt was made to compare the regression constants obtained with literature data. Apart from the interpretation formulas indicated in this study, it was found that there is a lack of data for soils corresponding to the Lublin aeolian loess. Significantly, also for cohesive soils of a different genesis there are few correlations of *q*_c_ with *I*_L_. In the literature, it is more common to find dependencies of *I*_*p*_ on *q*_c_, which, however, are only related to the grain size and mineralogical composition, without considering the natural water content of the subsoil. Meanwhile, this paper presents a strong dependence of *q*_c_ values on water content. The literature correlations between *I*_p_ and *q*_c_ values are characterized by a lower coefficient of determination, which for loess-like silty clay soil was 0.871^39^, while for various types of clay (with *I*_p_ in the range 4–77) R^2^ was determined to be 0.73^40^.

**Fig. 17:**
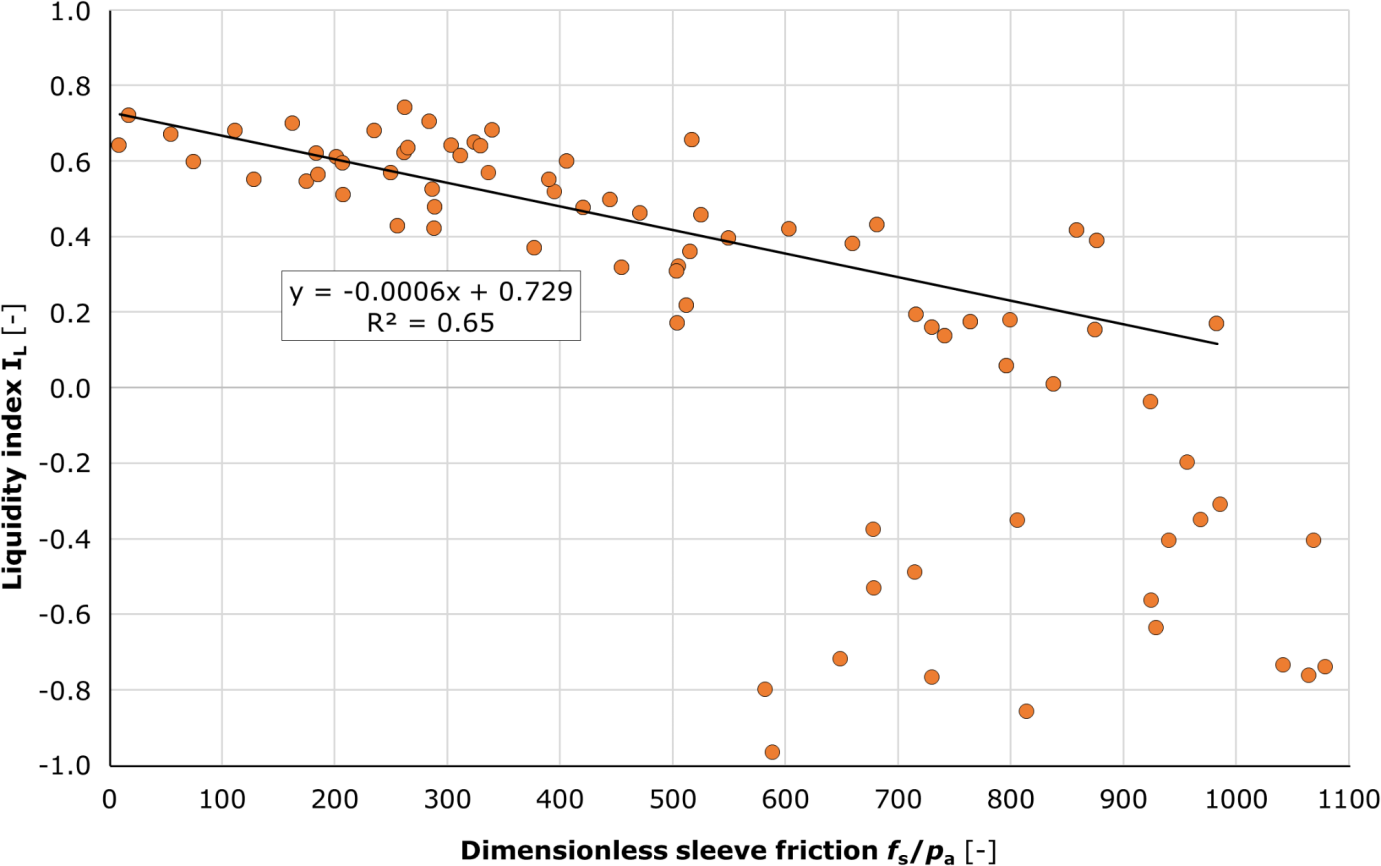
Chart of the (f_s_/p_a_) – I_L_ pairs distribution. A further addition to the study was the verification of a correlation between the dimensionless value of sleeve friction (*f*_s_/*p*_a_) and the liquidity index value. The laboratory-determined *I*_L_ values and the corresponding averaged *f*_s_/*p*_a_ values were plotted. A formula for a linear function has been derived, which represents a straight line approximating the points analysed in the *I*_L_>0 value range. However, it should be noted that the applicability of this function is limited to the fs values shown in the CPTU test at 5.5 MPa. It is inadvisable to employ values of *f*_s_/*p*_a_ in excess of 550 as a basis for estimating *I*_L_ values.

To complement the above analyses, the influence of the liquidity index on the sleeve friction was analysed. The graph (Fig. 17) compares the sleeve friction in dimensionless form *f*_s_/*p*_a_ with the liquidity index *I*_L_.

A discernible correlation was observed between the dimensionless sleeve friction value and the *I*_L_ value within the range of 0.0 to 0.8. Conversely, no such relationship was evident for *I*_L_ values less than 0.0. The plotted linear function for the *I*_L_ range of 0.8 to 0.0 was described by the following formula:

12 $$\:{I}_{\text{L}}=-0.0006\bullet\:{(f}_{\text{s}}/{p}_{a})+0.729\:[-]$$

The relationship between the dimensionless sleeve friction value and the *I*_L_ value is characterised by a coefficient of determination of R² = 0.65 with the covariance of -37.4 and the Pearson coefficient of 0.80. Based on the results obtained, it can be concluded that *I*_L_ can be reliably determined at *f*_s_/*p*_a_ < 550.

## Summary and conclusions

The paper presents the methodology of calibration analysis for the determination of the liquidity index using CPTU static sounding results. The research resulted in a valid formula as well as the nomogram for estimating the *I*_L_ value of the aeolian loess of the Nałęczów Plateau from the dimensionless cone resistance *q*_c_/*p*a. The function (9) derived from the analysis is recommended to estimate the liquidity index of the indicated loess in the range 0.0 ÷ 1.0. Semi-solid and solid states in the loess subsoil were noted when the cone penetration value exceeded 4.4. Stiff soil was anticipated for qc values between 3.25 and 4.4, while medium-soft soil was expected for values from 1.72 to 3.25. Soft soil was recognized with qc values ranging from 0.8 to 1.72. Cone resistance qc values from CPTU tests that fall below 0.8 suggest the existence of very soft soil. Moreover, a correlation was identified between the liquidity index and sleeve friction, which can be deemed a reliable indicator for the *f*_s_/*p*_a_ < 550 range.

In addition, the authors place particular emphasis on the fact, that cone resistance *q*_c_ rather than *I*_L_ should be used for determining geotechnical layers within a soil type. *I*_L_ can be used as a secondary index. Despite the high compliance of the formula, it should be remembered that static soundings only allows for a preliminary determination of the liquidity index. The appropriate value should be determined in laboratory tests.

## Data Availability

The data used to support the findings of this study are included within the article.
